# Effect of direct vs. indirect supervision on psychological outcomes of nursing students during clinical simulations: a quasi-experimental study

**DOI:** 10.3389/fmed.2025.1691882

**Published:** 2025-11-19

**Authors:** Azizah Alhaggas Albaqami, Homood Alharbi

**Affiliations:** 1 College of Nursing, King Saud University, Riyadh, Saudi Arabia; 2 Department of Nursing Education, College of nursing, Qassim University, Qassim, Saudi Arabia; 3 Department of Medical Surgical Nursing, College of Nursing, King Saud University, Riyadh, Saudi Arabia

**Keywords:** simulation training, education, nursing, faculty, anxiety, self-efficacy

## Abstract

**Background:**

Clinical simulation bridges the gap between theory and practice in nursing education. However, the optimal design specifically, direct observation or faculty presence, remains unsettled. This study compares the short-term effects of faculty presence during clinical-simulation re-demonstrations on student anxiety, self-confidence, and satisfaction, with the goal of informing more effective, student-centered simulation strategies.

**Methods:**

A pretest–posttest quasi-experimental design was implemented between January and April 2024 at a university in Saudi Arabia. A convenience sample of 138 senior Bachelor of Nursing students was divided into a control group (faculty member present in the simulation lab) and an intervention group (faculty members observing and evaluating students through a one-way mirror from the control room). Group allocation followed pre-existing class arrangements, and randomization was not performed. Data were collected using validated instruments: the State-Trait Anxiety Inventory (STAI, Forms Y-1/Y-2), the Student Satisfaction and Self-Confidence in Learning Scale (SCLS), and a demographic questionnaire.

**Results:**

Posttest results indicated that the intervention group demonstrated significantly lower state anxiety (*M* = 60.9) compared with the control group (*M* = 78.09; effect size *r* ≈ −0.22). The intervention group also reported higher self-confidence (*M* = 73.57) than the control group (*M* = 65.43; effect size r ≈ −0.07). Conversely, satisfaction scores were higher in the control group (*M* = 72.25) than in the intervention group (*M* = 66.75; effect size *r* ≈ −0.10). Among participants in the intervention group, female students exhibited significantly higher anxiety and lower satisfaction and self-confidence than male students (*p* < 0.05).

**Conclusion:**

The intervention enhanced students’ self-confidence and revealed gender-related differences in simulation learning. These findings underscore the need for tailored simulation strategies that address specific learning needs. Differentiated feedback and structured debriefings may strengthen confidence and reduce anxiety. Future research should explore faculty presence versus absence using larger samples and mixed-method designs to identify conditions optimizing learning and student affect.

## 1 Background

Student evaluation in clinical simulation supports skill development by giving direct feedback on clinical skills and decisions. This feedback helps identify strengths and areas needing improvement while building confidence in a safe, risk-free setting. Studying faculty presence during simulation assessments is crucial for developing evaluation strategies that enhance trainee skills. Direct observation is one method, where faculty in the simulation lab assess trainees in real time and offer immediate feedback (1). Faculty evaluate how students apply knowledge to practice and spot skill gaps. Observation through a one-way mirror allows faculty to watch without disrupting students. Structured debriefing after simulation helps students reflect and improve using faculty feedback (1). This method lets faculty observe skills without interfering. When faculty are present, observation and immediate feedback combine, allowing real-time monitoring, correction, and guidance. However, direct observation can increase anxiety and affect natural performance. Research notes this “observer effect” can obscure true reasoning and decision-making (2). Observation behind a one-way mirror reduces this bias, permitting more genuine responses but delaying feedback. Thus, debriefing after simulation is crucial; faculty use notes or recordings to give useful feedback without influencing behavior. Structured debriefings facilitate honest discussions of strengths and weaknesses, enabling students to link theory to practice and enhance their clinical skills (3). Whether feedback is immediate or delayed, following clear debriefing guidelines, including awareness of cognitive load, allows for fair and objective performance assessments (2). Structured debriefing overcomes the disadvantages of each method, ensuring reflection and learning are central (3). To sum up, faculty presence allows quick feedback but may distort performance, while a one-way mirror supports authentic responses, making structured debriefing vital for learning. Faculty training and consistent debriefing methods help both approaches provide fair assessments (3). Studying faculty presence can improve future clinical simulations in nursing by reducing anxiety, increasing confidence, critical thinking, judgment, satisfaction, and overall competence, all of which support better learner engagement and retention (4, 5).

## 2 Problem statement

There is a lack of empirical evidence on how the presence or absence of nursing faculty during clinical simulations affects nursing students’ anxiety, self-confidence, and satisfaction. Although high-fidelity simulators and observation enhancing features (control rooms, one-way mirrors) are increasingly adopted, few studies have examined whether faculty positioning either in the lab or in a control room modulates these student outcomes in the Saudi context. This evidence gap hinders evidence-based decisions about faculty placement in clinical simulations beyond personal preference. Therefore, robust research is needed to determine the impact of faculty presence on anxiety, confidence, and satisfaction among Saudi nursing students during clinical simulations.

## 3 Related literatures

This study aimed to investigate the state of anxiety, trait of anxiety, self-confidence, and satisfaction of nursing students to discover whether there is a significant difference between students who were evaluated during their re-demonstration with the presence of faculty member in the simulation lab (control group) and students who were evaluated when faculty are physically absent but observing through a one-way mirror of faculty members in the simulation room during clinical simulation (intervention group). The term re-demonstration refers to the act of demonstrating the nursing procedure again to ensure that students comprehend the skills learned, with the presence of a faculty member in the simulation room (control group) and students who were evaluated through a one-way mirror while the faculty member was in the control room (intervention group). The secondary objectives were to (i) assess whether there is a significant change in anxiety, self-confidence, and satisfaction among nursing students based on their selected group (control and intervention) and (ii) compare the quantitative differences in anxiety, self-confidence, and satisfaction levels among male and female nursing students. This study also posed the following research questions:

- Is there a statistical difference in students’ level of anxiety when a faculty member is present (control group) during clinical simulation compared to the same situation when the faculty are physically absent but observing through a one-way mirror (intervention group)?
- Is there a statistical difference in students’ level of satisfaction when a faculty member is present (control group) during clinical simulation compared to the same situation when the faculty are physically absent but observing through a one-way mirror (intervention group)?
- Is there a statistical difference in students’ level of self-confidence when a faculty member is present (control group) during clinical simulation compared to the same situation when the faculty members are physically absent but observing through a one-way mirror (intervention group)?
- Is there a statistical difference between male and female students in anxiety, self-confidence, and satisfaction when faculty are present (control group) during clinical simulation compared to the same situation when faculty are physically absent but observing through a one-way mirror (intervention group)?

The results of this study have important consequences for nursing education. Nursing educators can use this information to create learning environments that are more likely to result in successful student learning and reduce students’ anxiety and sense of isolation while being more effective at promoting student self-confidence.

The effect of faculty presence during clinical simulations has been a topic of considerable debate in nursing education. Horsley and Wambach examined the influence of faculty presence on students**’** anxiety, self-confidence, and clinical performance (6). Their study found that students**’** state anxiety, self-confidence, satisfaction, or clinical performance was not much changed by faculty presence (6). On pretest to posttest, students in the experimental group—where faculty members were present—showered a notable rise in state anxiety scores; the control group showed a notable drop in anxiety levels (7). This implies that even if the presence of faculty members might momentarily increase anxiety levels, it does not always negatively affect students**’** general level of satisfaction or clinical performance. Such findings underline the complexity of the link between faculty presence and student anxiety and the need of several elements influencing these effects. The literature indicates that employing a one-way mirror to observe students during clinical simulations is linked to improved performance in essential skills such as physical examinations, history taking, and diagnostic abilities. This method allows instructors to discreetly monitor students, enabling learners to participate in simulations without faculty physically present in the room, while still benefiting from faculty oversight and feedback (8). This implies that while still encouraging good learning, indirect faculty presence might help to lower anxiety.

Further studies have examined the effects of direct faculty observation during clinical simulations. A study looked at medical students**’** level of satisfaction both with and without direct faculty observation during clinical activities (9). Their results revealed that students favored clinical activities including direct faculty observation above those without faculty presence. With a rating of 3.02 rather than 2.67 for students who were not observed, the satisfaction scores for students under observation by faculty were noticeably higher (9). This suggests that even if direct observation might raise student anxiety because of the awareness of being watched, it also gives validation and support that boosts student satisfaction. These findings highlight how the advantages of faculty presence in simulations go beyond only lowering anxiety and can increase student satisfaction by providing real-time feedback and direction. Furthermore, another study emphasized the need of real-time comments in clinical simulations (1). They maintained that real-time feedback is crucial in enabling students to perform effectively in high-stakes situations, when exact execution and fast decision-making are required. For instance, faculty members can correct actions like drug distribution right away, so guiding students toward mastery of fundamental abilities. Reducing anxiety and encouraging a more confident and competent attitude to clinical tasks depends much on this feedback process. Therefore, helping students enhance their clinical skills and control anxiety depends on real-time feedback from faculty (1).

Gender differences in student anxiety, self-confidence, and satisfaction have been well-documented in clinical simulation studies. In a study investigated the degrees of state anxiety in male and female students and discovered that female students showed higher degrees of anxiety both before and during simulations (10). This implies that female students sometimes enter high-stress clinical simulations with more anxiety, which might influence their capacity to perform under pressure. This result is consistent with other studies which revealed that male students using high-fidelity simulation environments report more degrees of comfort and confidence (11). This more comfort in simulation environments could let male students negotiate clinical tasks with less anxiety, so improving performance and increasing self-confidence in these surroundings. While female students may benefit from strategies that help lower their anxiety and build confidence, male students may benefit from being encouraged to empathize and communicate more effectively. These gender variations in anxiety and self-confidence should thus be considered while designing simulation-based learning activities.

In terms of satisfaction, there is some variability in how male and female students report their experiences in clinical simulations. study revealed a modest but statistically significant variation in student satisfaction levels between men and women (12). Comparatively to their male counterparts, female students expressed more satisfaction (12). This implies that female students might find more value in the simulation experience even if their anxiety levels are higher, maybe because of the chances to hone their clinical skills or the encouraging comments given by the faculty. Romero-Castillo et al. discovered, however, that male and female nursing students had no appreciable variations in satisfaction, suggesting that gender variations in satisfaction may not always be as clear (13). These conflicting results imply that the design of the simulation, the type of feedback given, and the general learning environment may all affect how gender affects satisfaction in clinical simulations. Although gender could affect satisfaction, other elements such the students**’** past experiences or impressions of the classroom could also be very important.

Research has also explored differences in self-confidence between male and female nursing students in simulation labs. Male nursing students often report somewhat higher degrees of self-confidence than their female counterparts, according to Moreno-Cámara et al. (14). Men**’**s mean self-confidence score in their study was 4.63 ± 0.36; women**’**s mean score was 4.41 ± 0.83. The difference was not statistically significant, thus even if male students might feel somewhat more confident, the difference is not great enough to be of any practical relevance. On the other hand, a study highlighted the possible influence of simulation experiences on self-confidence by finding that, no notable variations were found in levels of satisfaction and self-confidence when differentiated by gender or age (15). These results imply that, depending on the feedback and experiences male and female students come across during the course of their education, simulation environments can significantly increase their confidence even if male students may have somewhat higher levels of self-confidence generally.

Based on the literature review, limited research addresses direct versus indirect supervised clinical simulations in nursing education, with most studies focusing on confidence, performance, satisfaction, and anxiety. Evidence on instructor presence remains mixed. In nursing education, scarce data limit generalizability regarding instructor effects on learning and performance. This study advances the literature by directly comparing direct and indirect simulations, offering empirical evidence on the psychological and educational impacts of instructor presence. Beyond its Saudi context, the findings contribute to global discussions on achieving balance between guidance and learner independence in simulation-based education. Future research should explore diverse supervision models and standardized methodologies to strengthen evidence in this field.

## 4 Conceptual framework

This study was guided by the Nursing Education Simulation Framework (NESF) and Cognitive Load Theory (CLT). The NESF, based on Pamela Jeffries’s Simulation Model (16), provides a comprehensive approach to studying student outcomes in clinical simulations. The framework includes teacher factors, student attributes, educational practices, simulation design characteristics, and outcomes (17). Within this framework, faculty presence and absence act as key variables shaping students’ emotional and cognitive experiences. Faculty presence may help reduce anxiety by providing real-time feedback and scaffolding learning, whereas faculty absence can heighten situational anxiety but foster independence, critical thinking, and self-regulation. These contrasting effects highlight the need to balance guidance with learner autonomy. Integrating CLT offers deeper insight into this interaction. CLT emphasizes optimizing cognitive resources by minimizing extraneous cognitive load while enhancing meaningful learning. Feedback in simulation thus functions both emotionally by alleviating anxiety and cognitively by directing attention toward essential tasks and preventing overload.

**FIGURE 1:**
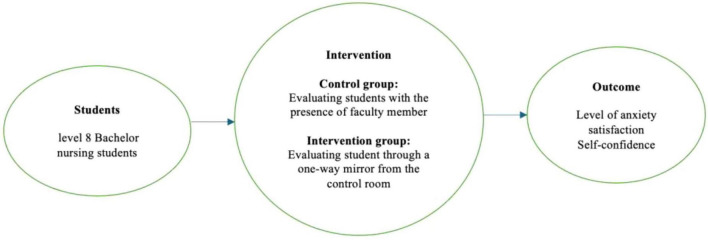
The study framework (adapted from the NLN/Jeffries simulation framework).

The combination of NESF and CLT links structure and process: NESF organizes the instructional design and learner variables, while CLT explains how these elements affect information processing and performance. This integration clarifies how faculty presence can both support learning and, if excessive, impede cognitive efficiency. Together, these frameworks establish the theoretical basis for examining how different supervision models influence anxiety, confidence, and satisfaction. They also emphasize the importance of designing simulations that balance emotional support and cognitive challenge to optimize student learning outcomes (Figure 1).

## 5 Methods

### 5.1 Design

To achieve the study objectives, we adopted a quantitative pre-post quasi-experimental design. In the medical literature, this is sometimes referred to as a controlled trial without randomization (18). This approach examined the impact of faculty member presence (independent variable) on students’ anxiety, self-confidence, and satisfaction levels (dependent variables) during clinical simulation in nursing education. Practical and ethical constraints of simulation-based nursing education informed our study design. Simulation-based nursing education utilizes role-playing or scenarios in controlled environments to simulate real-world clinical situations for educational purposes. As the literature (19) describes, we worked within established curricula and clinical simulation schedules. In these settings, we find it impractical to randomize participants into groups, as student groups typically form based on their clinical placements and course structures. Randomizing these natural groups could compromise the ecological validity of the simulation environment, which we define as the extent to which study conditions accurately reflect real-world practice. There are recognized challenges in implementing randomized controlled trials (RCTs) studies in which participants are randomly assigned to different groups to compare outcomes in simulation studies, due to logistical limitations and ethical considerations. RCTs offer methodological rigor but are not always practical in educational research. Maintaining the fidelity of real-world learning is critical (20). Simulation-based education, which uses activities or environments designed to closely replicate real clinical settings, mirrors authentic clinical interactions. Using a quasi-experimental design an approach that lacks random assignment but uses comparison groups preserves these dynamics and accurately represents students’ learning environments. In this context, randomization could introduce elements that interfere with the natural social and educational processes central to clinical simulation. The decision to forgo randomization aligns with recent simulation research. Quasi-experimental designs have been found to yield valid and comprehensive insights into faculty presence effects on learner outcomes (19, 20). By controlling confounders statistically and ensuring simulation realism, this study achieves strong internal validity and high ecological validity. These features enable a nuanced assessment of the effects of faculty presence on anxiety, self-confidence, and satisfaction, with findings applicable to real educational settings.

In this quasi-experimental study, pre-existing clinical groups were formed through the nursing program’s standard scheduling procedures. These groups were allocated to the intervention or control condition by randomizing groups rather than individuals. The randomization was done independently of the research team to minimize selection bias and maximize comparability. Although we used pre-existing groups and acknowledged potential baseline differences, the design aimed to balance groups wherever possible. To ensure the instrument’s suitability for this context, a pilot study involving 30 nursing students assessed the validity and reliability of the STAI and SCLS. Direct empirical evidence for these tools in this exact context is limited. However, piloting provides preliminary support, consistent with established instrument validation practices (21, 22). The quasi-experimental approach assumes the indicators of the experimental and comparison groups follow the same trajectory over time (23). The TREND statement was used to describe the study (24).

### 5.2 Study setting and sampling

**FIGURE 2:**
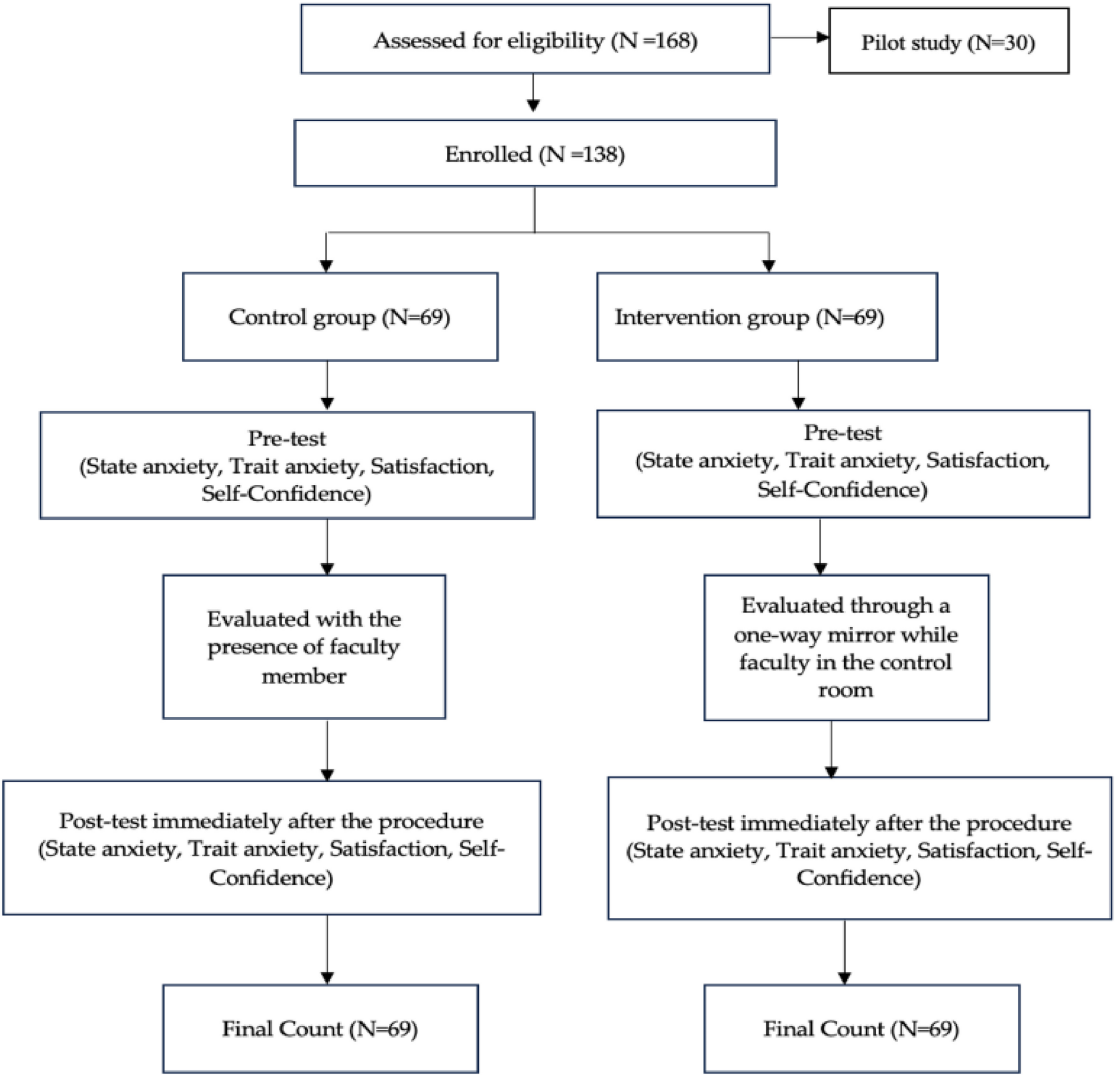
Participant selection process.

The sample consisted of 138 senior-level baccalaureate nursing students from a university in Saudi Arabia divided into an intervention group (*n* = 69) and a control group (*n* = 69). The sample size was calculated using an online priori sample size calculator, G*Power Version 3.1, using an alpha of 0.05 and a power of 0.80 with a medium effect size of 0.5. The minimum sample size for an independent sample *t*-test was 64 for each group and 67 for each group when using the non-parametric Mann-Whitney test, but a larger number of participants was chosen to account for student attrition and/or exclusion of students on anxiety medication, in psychological or behavioral therapy, or with previous work experience or who were not nursing students. The response rate was 98%, and there were no missing data because each item in the questionnaire was marked as required. To ensure homogeneity and avoid the cluster effect between the control and intervention groups, the students provided assurance that they were not on any anxiety medication or in psychological or behavioral therapy, and researchers verify that the control and intervention groups were similar in terms of demographics. The participant selection process is illustrated in Figure 2.

## 6 Data collection procedure

All data were collected via an online survey with three questionnaires: (1) demographics, (2) anxiety via STAI Forms Y-1/Y-2, and (3) satisfaction/self-confidence via SCLS. Participants were pre-assigned by the faculty into small groups (≤16) and instructors and nursing students were allocated to clinical simulation labs before the semester, with no researcher control over faculty selection or group formation as part of the curriculum. Data were collected January–April 2024. Pretest assessed state/trait anxiety and satisfaction/self-confidence; participants then engaged in a 35**–**40-min high-fidelity clinical simulation relevant to their course. The simulation scenarios used in this study were part of the students**’** clinical skills curriculum and were utilized in accordance with the International Nursing Association for Clinical Simulation and Learning (INACSL) Healthcare Simulation Standards of Best Practice (25). Except for the presence of the nursing staff, the atmosphere within the clinical simulation area was the same for both groups. Throughout the study, the researcher ensured that the nursing faculty members’ appearances, attitudes, and locations within the simulation room were the same for all groups. Following the clinical simulation re-demonstration, the researchers assessed the students’ state and trait anxiety levels using STAI Forms Y-1 and Y-2, and students’ satisfaction and self-confidence levels using the SCLS as a posttest which was administered by the same faculty member at the end of the simulation session. To reduce bias, researchers attended every simulation to ensure efficient survey administration. Faculty members did not participate in data analysis, and responses were collected anonymously to minimize social desirability bias. These measures align with established bias-reduction practices in survey research and help preserve data reliability (26). Moreover, by keeping faculty members separate from the data analysis process, the risk of subjective bias affecting the interpretation is reduced—a strategy that is endorsed by research advocating for objectivity in surveys dealing with sensitive topics (27). Collecting responses anonymously is widely acknowledged as an effective strategy to reduce social desirability bias. This approach fosters an environment in which participants are more likely to answer honestly, free from concerns about judgment or potential negative consequences (27, 28).

## 7 Study intervention

Both groups comprised nursing students who completed the same core clinical skills training, including mannequin-based simulations. They were evaluated before and after the clinical re-demonstration. In the intervention group, faculty watched via a one-way mirror from the control room; the control group was observed with the instructor present in the lab to reflect real-world conditions. While the control room staff could communicate through a microphone, researchers aimed to minimize contamination by keeping interventions simple and providing clear information about the study. Sessions were closely supervised to ensure protocol adherence, and any deviations would be documented, though none occurred. We acknowledge that even observational presence in the control group could influence outcomes and suggest future studies employ stricter monitoring or blinding. To reduce cross-group contamination, sessions were scheduled at different times and participants were instructed not to discuss the simulation content with peers until study completion.

## 8 Measuring implementation fidelity

Fidelity was examined through the lens of adherence, exposure, quality of delivery, participant responsiveness, and program differentiation, which were identified by Dane and Schneider and supported by Dane and Schneider (29) and Mihalic (30). These dimensions were used to investigate the impact of faculty member presence versus absence on nursing students’ levels of anxiety, self-confidence, and satisfaction during clinical simulation in nursing education. In this study, adherence is ensured by issuing detailed written guidelines to participants and faculty, with the researcher observing and monitoring the simulation to confirm faithful implementation; the researchers also attend sessions to verify adherence. Exposure is controlled so that both groups (control and intervention) undergo the same number of simulations in identical environments, using the same equipment and durations. Quality of delivery is standardized by training intervention-group faculty in advance to provide consistent oversight and support, with procedures themselves standardized to maintain uniform delivery. Participant responsiveness is assessed through engagement measures, using self-reports (SSCL and STAI-AD) and demographic data, complemented by an observation checklist during simulations. Finally, program differentiation is maintained by keeping the control group with a faculty presence and the intervention group without, making presence the sole differentiating factor between groups.

## 9 Instrument with validity and reliability

### 9.1 Demographics

The pretest demographic questionnaire, developed by the researcher, included five self-reported items on gender, age, experience, and use of anxiety medications or therapy. It served to verify inclusion/exclusion criteria but was not statistically controlled for in the main analyses, due to the sample’s relative demographic homogeneity. Specifically, no participants had prior behavior-therapy experience, and age/experience variability was minimal, making these factors unlikely confounders for anxiety, self-confidence, and satisfaction.

### 9.2 STAI-AD

STAI Forms Y-1 and Y-2 were developed by Charles Spielberger, R.L. Gorsuch, and R.E. Lushene. This instrument has been proven to be a reliable and valid measure of state and trait anxiety in adults (31). and has high internal consistency, with Cronbach**’**s alpha coefficients ranging from 0.86 to 0.95 (31). It consists of 40 items that require responses on a four-point Likert-type scale. It was administered to the students before and after the simulation session to measure their anxiety levels.

### 9.3 The student satisfaction and self-confidence in learning instrument

The SCLS was developed by the NLN in 2006, and its reliability and validity have been thoroughly examined. The SCLS ranged from 1 (strongly disagree) to 5 (strongly agree). The Cronbach**’**s alpha was 0.94 for satisfaction and 0.87 for confidence (32). Nine simulation specialists reviewed the tool to ensure content validity (32). The scale has been deemed trustworthy and valid for measuring students’ learning satisfaction and self-confidence, especially in high-fidelity simulation methods (33). This 13-item instrument requiring responses on a 5-point Likert scale was administered to nursing students to measure their levels of satisfaction and self-confidence in learning.

These tools are well suited for this context because clinical simulations can provoke anxiety from performance pressure. The STAI captures both state (immediate) and trait anxiety, enabling detection of situational and personal factors affecting performance and learning. The SCLS assesses self-confidence and satisfaction, shedding light on how students cope with anxiety and simulation experiences. Together, these measures offer a concise, comprehensive view of factors influencing anxiety, self-confidence, and satisfaction during clinical simulations.

Although the original validation of these instruments’ dates back several decades, our study took proactive measures to ensure their relevance and accuracy in the contemporary Saudi context. Specifically, while the SCLS has previously demonstrated robust psychometric properties in studies involving Saudi nursing students (34), we further established its applicability by convening pilot testing with a representative subset of our target population (*N* = 30). For the STAI, although direct validation data in Saudi settings is less abundant, our pilot study included rigorous reliability analyses to confirm acceptable levels of internal consistency and cultural appropriateness before its widespread application in our research (Table 1). These steps are consistent with best practices in instrument validation, which advocate for ongoing evaluation of reliability and validity, particularly in new cultural or educational contexts (35).

**TABLE 1:** Summary of reliability coefficient for the tools.

Tool	Cronbach’s alpha	N of items
Tool Y1	0.832	20
Tool Y2	0.883	20
Satisfaction	0.887	5
Self-confidence	0.942	8

## 10 Data analysis

The data gathered in this study were analyzed using Statistical Package for Social Sciences (SPSS) version 29 software. Descriptive statistics were used to describe the characteristics of study participants. Minimum, maximum, median, and interquartile ranges, and effect size were used to describe the target variables. Chi-square tests were used for categorical variables such as variations in sample characteristics between the intervention and comparison groups. The Shapiro–Wilk and Kolmogorov–Smirnov tests were conducted to determine the normality of the target variables. The distribution of the target variables was significantly different from normality with a *p* < 0.05. It has been confirmed that data are more likely to deviate from normality when their skewness and kurtosis are not close to zero, regardless of the sample size (36), as was the case in this study. Thus, a non-parametric method called the Mann-Whitney U test was used to analyze the data. A non-parametric test was used because it does not assume normality in data distribution and is ideal for comparing the distributions of two independent groups, such as pretest and posttest differences between the control and intervention groups in state anxiety, trait anxiety, satisfaction, and self-confidence. Although every item on the questionnaire was required, which ensured that there was no missing data for any completed survey, participants who did not finish the entire questionnaire were excluded from the analysis. Ultimately, only fully completed surveys were incorporated into the final dataset, and none of the students omitted the survey entirely. Although group assignments were predetermined by faculty, no significant differences in baseline characteristics were observed between groups.

## 11 Results

### 11.1 Pilot study results

Before conducting the main study, a pilot study involving 30 students from the same population was conducted (using the same data collection procedure as in the main study) to test the validity and reliability of the data collection tools and to assess the feasibility of the study (Table 1). No issues were noted, and all instruments were found to be reliable (Cronbach’s alpha > 80). According to Polit and Beck, reliability coefficients are commonly utilized in a variety of contexts and vary from 0.00 to 1.00 but are not negative in value. The higher the coefficient, the more reliable are the results (18).

### 11.2. Sample characteristics

The study sample (Table 2) consisted of 65.2% female and 34.8% male students, consistent with the composition of nursing students in Saudi Arabia. The 22–24-years age group was the most prevalent at 55%, with those aged between 19 and 21 years comprising the remaining 45%. None of the participants reported having previous experience, being on medication to treat anxiety, or receiving psychological therapy for anxiety. This experiential and status homogeneity of treatment is essential for isolating the effects of the intervention.

**TABLE 2:** Characteristics of study samples.

Characteristic	Group	Intervention	Control	Total
Gender	Male	24	24	48
	Female	45	45	90
Age	19**–**21	31	31	62
	22**–**24	38	38	76
	25 and above	0	0	0
Previous clinical experience	Yes	0	0	0
	No	69	69	138
Medication to treat anxiety	Yes			0
	No	69	69	138
Therapy to treat anxiety	Yes	0	0	0
	No	69	69	138

To examine the demographic characteristics (Table 2), the chi-square test of independence for categorical data was performed. This test is suitable for comparing the frequencies between two independent sets of categorical data. A chi-square test of independence for gender was unnecessary because gender distribution was the same in all groups, with 24 males (34.8%) and 45 females (65.2%) in each group. A chi-square test was conducted on the age categories, and the result was *p* = 1.00, indicating that there is no significant difference in age distribution between the intervention and control groups. None of the participants in either group had prior clinical experience, were on anxiety medication, or were receiving psychological therapy to manage anxiety; therefore, no statistical test was required. The intervention and control groups were equivalent in all measured demographic parameters. This indicates that the groups were well-matched, which is critical to the validity of the quasi-experimental design. No statistical testing revealed any significant differences between the groups as they were completely matched for all characteristics. Thus, homogeneity among participants was ensured at the beginning of the study.

Table 3 presents quartile statistics for state anxiety, trait anxiety, satisfaction, and self-confidence across different testing conditions: pre-test and post-test for both the control and intervention groups. For the control group, the median state anxiety was higher (Med = 2.1) in the pre-test to (Med = 2.05) in the post-test, indicating an increase in anxiety levels. Trait anxiety showed an increase in the median from (Med = 1.6 to 2.3), while the median satisfaction for the control group decreased from 4.6 to 3. 8. Self-confidence increased slightly from a median of 4.25 to 4.375. These trends are consistent with observations that simulations and similar educational interventions can reinforce positive psychological outcomes in nursing students by increasing satisfaction and confidence while reducing anxiety (37, 38). In contrast, the intervention group exhibited more pronounced changes. State anxiety decreased from a median of 2.05 to 1.95, and trait anxiety decreased from 2.3 to 2.2, suggesting effective intervention outcomes in reducing anxiety levels. Notably, satisfaction increased significantly from a median of 3.6 to 4.0, and self-confidence also improved from 3.875 to 4.0, indicating that the intervention positively impacted these psychological constructs. Overall, these results suggest that the intervention was effective in reducing anxiety and enhancing self-confidence and satisfaction among participants when compared to the control group. These modifications suggest that the intervention has had a beneficial effect by mitigating anxiety and promoting self-confidence and satisfaction among the students. Such trends are in line with findings where simulation-based educational interventions have been associated with improved self-confidence and satisfaction, as well as decreased anxiety, emphasizing the role of these pedagogical strategies in enhancing the overall learning experience (37–39). Quartile distributions show the control group maintained high satisfaction and self-confidence, while the intervention group reduced both state and trait anxiety and improved satisfaction and self-confidence from pretest to posttest. This improvement underscores the potential efficacy of targeted educational interventions in positively influencing the psychological and educational outcomes of nursing students, thereby supporting the integration of simulation-based learning methods in nursing education curricula (37–39).

**TABLE 3:** Descriptive statistics of the target variables.

			Quartiles		
Pretest control	Minimum	Maximum	First	Median	Third
State of anxiety	1	3.05	2.1	2.1	2.575
Trait of anxiety	1	2.85	1.6	1.6	2.45
Satisfaction	1	5	4	4.6	5
Self-confidence	1	5	4	4.25	4.25
**Posttest control**					
State of anxiety	1	3	2.05	2.05	2.525
Trait of anxiety	1	2.85	2.1	2.25	2.45
Satisfaction	1	5	3.8	3.8	4
Self-confidence	1	5	4	4.375	4.625
**Pretest intervention**					
State of anxiety	1.15	2.85	1.9	2.05	2.5
Trait of anxiety	1.1	3.1	1.75	2.3	2.45
Satisfaction	2	5	3.2	3.6	4
Self-confidence	2.38	5	3.75	3.875	4
**Posttest intervention**					
State of anxiety	1	3.1	1.8	1.95	2.5
Trait of anxiety	1	3.05	2.2	2.2	2.55
Satisfaction	1	5	4	4	5
Self-confidence	1	5	4	4	4.625

Table 4 summarizes the descriptive statistics for all key study variables at both pre-test and post-test time points. For each variable, the mean and standard deviation, as well as minimum, maximum, and percentile values, are reported for the total sample (*N* = 138): State of anxiety and trait of anxiety scores remained relatively stable from pre-test (*M* = 2.21, SD = 0.46 and *M* = 2.07, SD = 0.48, respectively) to post-test (*M* = 2.18, SD = 0.48 and *M* = 2.13, SD = 0.46, respectively). Satisfaction and self-confidence showed slight increases from pre-test (*M* = 3.95, SD = 0.90 and *M* = 3.85, SD = 0.77) to post-test (*M* = 4.25, SD = 0.94 and *M* = 4.01, SD = 0.81). The control/intervention group variable remained constant, reflecting group allocation.

**TABLE 4:** Descriptive statistics for main study variables (pre-test and post-test).

Variable	Time point	N	Mean	SD
State of anxiety	Pre-test	138	2.21	0.46
State of anxiety	Post-test	138	2.18	0.48
Trait of anxiety	Pre-test	138	2.07	0.48
Trait of anxiety	Post-test	138	2.13	0.46
Satisfaction	Pre-test	138	3.95	0.90
Satisfaction	Post-test	138	4.25	0.94
Self-confidence	Pre-test	138	3.85	0.77
Self-confidence	Post-test	138	4.01	0.81
Control/intervention group	Pre-test	138	1.50	0.50
Control/intervention group	Post-test	138	1.50	0.50

### 11.3 Levels of state anxiety, trait anxiety, satisfaction, and self-confidence

The Mann-Whitney U test compared intervention and control groups on pretest and posttest measures of state anxiety, trait anxiety, satisfaction, and self-confidence.

Table 5 shows that in the pretest, the intervention group had lower mean ranks than the control for state anxiety (*M* = 62.51; *p* = 0.038), satisfaction (*M* = 53.07; *p* = 0.000), and self-confidence (*M* = 59.25; *p* = 0.002), but higher for trait anxiety (*M* = 78.68; *p* = 0.007). In the posttest, they showed higher trait anxiety (*M* = 79.82; *p* = 0.002) and lower state anxiety (*M* = 60.91; *p* = 0.011). Pretest differences were significant across all measures, while posttest differences remained in state and trait anxiety.

**TABLE 5:** Results of Mann-Whitney U tests.

Variable	Control (mean rank)	Intervention (mean rank)	Mann-Whitney U	Z	Effect size r	Asymp. Sig. (2-tailed)
**Pretest**						
State anxiety	76.49	62.51	1898.000	−2.070	−0.176	0.038
Trait anxiety	60.32	78.68	1747.000	−2.721	−0.232	0.007
Satisfaction	85.93	53.07	1247.000	−4.907	−0.418	0.000
Self-confidence	79.75	59.25	1673.000	−3.056	−0.260	0.002
**Posttest**						
State anxiety	78.09	60.91	1788.000	−2.553	−0.217	0.011
Trait anxiety	59.18	79.82	1668.500	−3.066	−0.261	0.002
Satisfaction	72.25	66.75	2191.000	−0.856	−0.073	0.392
Self-confidence	65.43	73.57	2099.500	−1.219	−0.104	0.223

Posttest analyses demonstrated a shift in outcome patterns. The intervention group continued to exhibit significantly lower state anxiety (*p* < 0.05) yet maintained higher trait anxiety levels compared to the control group, indicating that the intervention was more effective in reducing situational than dispositional anxiety. Previous differences in satisfaction and self-confidence were no longer statistically significant, suggesting positive improvement over time. However, baseline disparities between groups may have influenced these outcomes, potentially confounding the observed posttest effects. To account for this, future analyses might consider adjusting for baseline scores using techniques such as ANCOVA (40, 41). Mann-Whitney U test effect sizes (r) indicated predominantly small to moderate differences between groups at pretest and posttest. For state and trait anxiety, effects ranged from −0.176 to −0.261, with the intervention group generally showing lower anxiety. Pretest satisfaction showed a moderate effect (*r* = −0.418), with the control group reporting higher satisfaction. Self-confidence differences were small to moderate (*r* = −0.260 pretest; *r* = −0.104 posttest), and posttest improvement in the intervention group was not statistically significant. Overall, despite some significant findings (*p* < 0.05), the practical impact was limited except for the moderate pretest satisfaction effect.

We analyzed outcomes by gender to assess if the intervention’s impact on anxiety, self-confidence, and satisfaction remained consistent between male and female students, or if gender-specific variations influenced these psychological and educational responses. This additional analysis bolsters the generalizability of our findings and aids in identifying and addressing any potential gender-related disparities within nursing education. Table 5 presents the results of the Mann-Whitney U tests comparing male and female in control groups based on four variables: state anxiety, trait anxiety, satisfaction, and self-confidence. Male students had a significantly lower state anxiety (*M* = 22.50) than female (*M* = 41.67), as was also the case for trait anxiety (*M* = 21.00; *M* = 42.47); however, male had significantly higher satisfaction (*M* = 52.50) than female (*M* = 25.67) as well as higher self-confidence (*M* = 47.50; *M* = 28.33). For all variables, the differences between males and females were significant (*p* < 0.05). The negative Z-scores for all four variables indicated a consistent difference between male and female nursing students during the clinical simulation.

Table 6 presents the results of the Mann-Whitney U tests comparing male and female participants in the pretest intervention group across the four variables. Males (*M* = 23.81) had significantly lower state anxiety than females (*M* = 40.97). They (*M* = 19.15) also had significantly lower trait anxiety than females (*M* = 43.46). Males (*M* = 18.52) reported significantly lower satisfaction than females (*M* = 43.79) as well as significantly lower self-confidence (*M* = 27.92) than females (*M* = 38.78). All four *p-*values were less than the predetermined alpha level of 0.05, indicating that all results were considered statistically significant.

**TABLE 6:** The results male and female control groups.

Variable	Gender	Pretest (control)	Mann-Whitney U	Z	Asymp. Sig. (2-tailed)
State anxiety	Male	22.50	240.000	−3.916	0.000
	Female	41.67			
Trait anxiety	Male	21.00	204.000	−4.401	0.000
	Female	42.47			
Satisfaction	Male	52.50	120.000	−5.661	0.000
	Female	25.67			
Self-confidence	Male	47.50	240.000	−3.931	0.000
	Female	28.33			

Table 7 presents the results of Mann-Whitney U tests comparing male and female groups on four variables: state anxiety, trait anxiety, satisfaction, and self -confidence Males have significantly lower state anxiety (*M* = 24.50) than females (*M* = 40.60), significantly lower trait anxiety (*M* = 18.50) than females (*M* = 43.80), significantly lower satisfaction (*M* = 23.00) than females (*M* = 41.40), and significantly higher self-confidence (*M* = 50.50) than females (*M* = 26.73). For all variables, the differences between males and females were statistically significant (*p* < 0.05). The negative Z-scores for all four variables indicate a consistent difference between males and females across the control group.

**TABLE 7:** The results for the male and female intervention groups.

Variable	Gender	Pretest (inter vention)	Mann-Whitney U	Z	Asymp. Sig. (2-tailed)
State anxiety	Male	23.81	271.500	−3.455	0.001
	Female	40.97			
Trait anxiety	Male	19.15	159.500	−4.903	0.000
	Female	43.46			
Satisfaction	Male	18.52	144.500	−5.075	0.000
	Female	43.79			
Self-confidence	Male	27.92	370.000	−2.191	0.028
	Female	38.78			

Table 8 presents the results of the Mann-Whitney U tests comparing male and female participants in the posttest intervention group across the four variables. Males (*M* = 22.83) had significantly lower state anxiety than females (*M* = 41.49). They (*M* = 24.85) also had significantly lower trait anxiety than females (*M* = 40.41). Males (*M* = 53.83) reported significantly higher satisfaction than females (*M* = 24.96) as well as significantly higher self-confidence (*M* = 50.88; *M* = 26.53). These results indicate substantial gender differences in all four variables after the intervention, with males consistently showing more favorable outcomes (lower anxiety, higher satisfaction, and higher self-confidence) than females. The differences were statistically significant for all variables, suggesting that the intervention may have had different effects on nursing students by gender (Table 9).

**TABLE 8:** The results for the male and female control groups.

Variable	Gender	Posttest (Control)	Mann-Whitney U	Z	Asymp. Sig. (2-tailed)
State anxiety	Male	24.50	288.000	−3.275	0.01
	Female	40.60			
Trait anxiety	Male	18.50	144.000	−5.159	0.000
	Female	43.80			
Satisfaction	Male	23.00	252.000	−3.803	0.000
	Female	41.40			
Self-confidence	Male	50.50	168.000	−4.928	0.000
	Female	26.73			

**TABLE 9:** The results for male and female intervention groups.

Variable	Gender	Posttest (inter vention)	Mann-Whitney U	Z	Asymp. Sig. (2-tailed)
State anxiety	Male	22.83	248.000	−3.827	0.000
	Female	41.49			
Trait anxiety	Male	24.85	296.500	−3.169	0.002
	Female	40.41			
Satisfaction	Male	53.83	88.000	−6.094	0.000
	Female	24.96			
Self-confidence	Male	50.88	159.000	−5.034	0.000
	Female	26.53			

## 12 Discussion

This study is the first to explore the impact of faculty presence vs. physically absent but present in the control room through a one-way mirror on nursing student anxiety, self-confidence, and satisfaction during clinical simulations in Saudi Arabia. While the intervention group who lacked faculty presence reported higher trait anxiety and lower levels of state anxiety, satisfaction, and self-confidence, the control group showed higher levels of these during the pretest phase. These findings line up with earlier studies looking at faculty presence during clinical simulations. For example, Barcelona et al. (42) noted increased anxiety among graduate students assessed in front of a teacher. Their results imply that students’ awareness of being watched in real-time can raise tension and stress since they feel more pressure to perform well, which relates with the high state anxiety shown in the control group of the present study. Likewise, a study underlined that real-time evaluation could divert students from their studies since they might cause them to become unduly preoccupied with the assessment instead of the work at hand (43, 44). These results suggest that although direct observation in simulations might cause increased anxiety, it could also result in positive effects including validation and teacher support.

On the other hand, the higher trait anxiety of the intervention group could point to a different psychological profile whereby students might have a general inclination toward anxiety, which might not be immediately addressed by the absence of faculty. While state anxiety is usually brought on by situational stresses, such real-time evaluations, trait anxiety is a steadier, personality-driven component of anxiety. Lack of instantaneous feedback could have contributed to the intervention group’s insecurity, so reducing self-confidence and satisfaction. This is consistent with studies which revealed that instructor presence might foster a supportive environment, thus boosting students’ confidence in their abilities even in cases of anxiety (45). However, the intervention group may have lacked this support, impacting their self-confidence and overall satisfaction with the simulation experience.

Interpreting these results within the Nursing Education Simulation Framework (NESF) provides theoretical clarity. The NESF emphasizes the facilitator’s role in ensuring psychological safety, guiding reflection, and linking learning experiences to clinical competence. The observed group differences suggest that faculty presence directly contributes to these elements by fostering learner support and constructive feedback loops. Conversely, faculty absence may improve situational comfort but at the cost of reduced emotional engagement and satisfaction. Nonetheless, the study’s non-randomized design limits causal inference; baseline disparities between groups could have influenced both pretest and posttest outcomes.

Un-expected results of this study were the differences in self-confidence and satisfaction between the intervention and control groups. The control group claimed higher levels of self-assurance and satisfaction even though they had more state anxiety. Faculty participation in simulations may explain this. Faculty validation reduces stress. Student success is boosted by real-time teacher feedback, even for anxious students. This feedback system encourages appropriate behavior and allows instantaneous corrections, helping students become more self-aware. Without faculty, the intervention group lacked this support. Their confidence and satisfaction may have suffered. Although their anxiety decreased, their lack of faculty direction may have hampered their performance. This supports Elendu et al. (1), who claimed real-time comments reduce anxiety and boost self-confidence. Gender differences in the simulation environment were another important research finding. Female intervention students reported lower self-confidence, anxiety, and satisfaction than male students. Current studies show that women, especially in performance-related events, feel more anxious (10). Female students’ lower self-confidence and satisfaction are concerning because these factors can affect their clinical performance and academic experience. These results imply that more study is required to investigate how gender shapes students’ experiences in clinical simulations (46, 47). Possible confounding factors warrant careful consideration. Variations in prior clinical experience, personality traits, or exposure to simulation could shape how students respond to observable versus indirect faculty involvement. Cultural dynamics in Saudi education also play a role: instructors often occupy positions of authority that carry both evaluative and mentorship implications. Thus, faculty presence may simultaneously elicit anxiety and serve as an emotional anchor—an ambivalence unique to hierarchical educational contexts. Future research should explore these sociocultural influences to better contextualize the relationship between faculty presence, perception, and performance.

This study found that men in the control and intervention groups had higher self-confidence and satisfaction than women. Male students in the control group had higher self-confidence, suggesting they thought they were better at simulations. These findings support by other studies who found that clinical simulations boost male students’ self-confidence (14, 15). However, female students reported lower self-confidence and satisfaction, suggesting they may be more affected by simulated performance pressure. Female students may need more personalized feedback or mentoring programs to boost their confidence and reduce anxiety in high-pressure situations. Clinical simulations should address gender-specific anxiety, self-confidence, and satisfaction issues, according to the study. Nursing schools should foster an inclusive, supportive environment for all students. Giving female students personalized feedback that highlights their strengths and offers growth opportunities may reduce stress and boost confidence. Mentorship programs that pair female students with experienced nurses may also provide role models and clinical management advice. Mentors can help female students overcome their unique stressors. Stress management seminars and resilience-building workshops could also help gender-based anxiety and self-confidence differences. These interventions may help female students handle clinical simulation pressures. These strategies help nursing programs create a more welcoming learning environment. This would boost female students’ academic performance and simulation satisfaction, improving outcomes for all students. In support of our finding, previous research in healthcare and dental education has documented similar trends, suggesting that gender-based socialization and evaluative pressures may contribute to higher anxiety levels and lower self-confidence among female students (48). These differences could potentially impact clinical decision-making and overall educational outcomes. Future studies could build on our findings by investigating how these affective dimensions interact with clinical performance and what targeted interventions might mitigate the negative effects. Such research would help in developing tailored support strategies to better prepare all students for the demands of clinical practice.

Although the study provides insightful analysis of the effects of faculty presence and gender variations in clinical simulations, more study is required to investigate these conclusions more thoroughly. Future studies could examine how different types of feedback (e.g., written vs. verbal) influence anxiety, self-confidence, and satisfaction among students. Furthermore, the creation of adaptive simulation models considering psychological profiles and personal needs could assist to solve the different degrees of anxiety and self-confidence noted in this work. Research on gender-specific interventions in clinical simulations should last as well to produce more fair learning settings for male and female students. Comparable gender-related differences have been reported in healthcare and dental education, suggesting that the phenomenon extends beyond nursing and may be influenced by broader societal and cultural factors. Integrating structured psychosocial support and adaptive feedback mechanisms could therefore enhance equity and inclusivity within simulation-based education. Additionally, future studies should examine the interplay between gender, cultural expectations, and academic background using longitudinal and randomized approaches to validate these findings across diverse cohorts.

Although this study offers valuable insights into emotional and attitudinal aspects of simulation learning, several limitations should be noted. The non-randomized design limits causal inference and may have introduced selection bias, as baseline differences in anxiety, confidence, or satisfaction could have influenced outcomes. Uncontrolled factors such as personality traits, coping styles, or cultural values may also have affected responses to faculty presence. Future research should employ randomized and mixed-method designs to examine feedback modalities, cultural influences, and gender-based differences in shaping simulation learning experiences.

## 13 Strengths and limitations of the work

### 13.1 Strengths

The strength of this study lies in the confirmation of the reliability and validity of the instruments used in a pilot study conducted with 30 bachelor-of-nursing students before the commencement of the main study. The pilot study revealed that the participants understood the assessment instruments. Their reliability was likewise satisfactory (Cronbach alpha > 0.80) (18), and all of the tools used in this study were valid and reliable as their validity and reliability were confirmed decades prior. While the instruments’ original validity was established in past decades, we have undertaken recent validation steps including pilot testing to ensure their reliability and validity for contemporary nursing education research. This approach aligns with best practices in instrument validation, which recommend ongoing evaluation to maintain both reliability and validity in changing research contexts. The study achieved a response rate of 98%, which was considered a significant strength of the study and its finding. In addition, the outcomes of this study will address an existing gap in the literature concerning the differential effects of having a faculty member physically present versus physically present in the control room during clinical simulations on nursing students’ anxiety, self-confidence, and satisfaction. Although previous investigations have examined a variety of simulation design components, there is scarce and inconclusive evidence regarding how the physical presence of faculty during summative simulation assessments impacts these psychological and educational outcomes. This lack of clarity extends to the debate on whether faculty should remain inside the simulation room or observe remotely in order to optimize student learning and well-being during high-stakes assessments (7). Therefore, the primary objective of this study is to generate empirical data that will help determine best practices for faculty positioning during summative simulation exercises in nursing education.

### 13.2 Limitations

The current study has limitations that may help future researchers understand and improve their studies. A key limitation of this study was that it did not account for confounding factors. Confounding factors are unmeasured variables that may influence cause and effect. In the present study, students**’** anxiety levels may have been influenced by factors other than the presence of faculty members. For example, students’ individual factors may explain the changes in anxiety levels between the control and intervention groups. In addition, the levels of self-confidence may also decrease or increase based on factors such as how nursing students were prepared for the simulation and whether they felt that they were equipped to perform well in the clinical simulation. Other confounding factors were related to the satisfaction levels. For example, in addition to the presence of a faculty member, the mood of the students and their interactions during the simulation may also affect their performance and satisfaction. Failure to account for these confounding factors may lead to biased results and erroneous conclusions. while we discussed the potential influence of confounding variables such as prior simulation experience and academic background, we did not perform specific statistical adjustments or stratified analyses in this study. We recognize this as a limitation and recommend that future research incorporate such methods to better isolate the effects of faculty presence versus absence for example using ANCOVA as an additional statistical analysis. While faculty were trained and familiar with the tool, we acknowledge that a formal assessment of inter-rater reliability (e.g., calculating an intraclass correlation coefficient) was not conducted as part of this study. We recognize the importance of formally documenting inter-rater reliability to further ensure the rigor and consistency of faculty evaluations, as recommended in the simulation education literature. The convenience sample approach, single institution, same academic level, and nursing course (homogeneity) may limit the nationwide generalizability of this study. Therefore, future studies should consider multi-site studies featuring a larger sample size and a student pool from different disciplines to improve the generalizability of the findings. Our study utilized a convenience sampling method by recruiting participants from a specific academic institution, following a similar approach to previous studies (49, 50). No additional measures such as stratified sampling or weighting were taken to ensure the sample fully represented nursing students throughout Saudi Arabia. We acknowledge that this sampling method may restrict the generalizability of our results. Nonetheless, by detailing the demographic characteristics of our sample, we allow readers to contextualize and critique the study**’**s potential limitations. Future studies should incorporate probabilistic sampling techniques to better capture the diversity of the nursing student population in Saudi Arabia. The study has a quasi-experimental design, but its cross-sectional elements complicate the assessment of causal relationships (51). The study results relied on self-reported questionnaires, which may have biased online survey responses. This limitation was addressed by ensuring the anonymity and encourage honest responses from the participants. Moreover, the number of prior studies on this topic that could be used to compare the findings of the present study was limited.

## 14 Implications

### 14.1 Broader implications

These findings can be contextualized within nursing education by differentiating state and trait anxiety. State anxiety reflects a temporary response to immediate stressors (e.g., clinical simulations) and can motivate preparation and performance when kept at low-to-moderate levels. By contrast, trait anxiety is a stable predisposition to heightened anxiety across contexts and tends to produce enduring negative effects, impairing cognitive functions such as critical thinking and decision-making. This distinction is essential because interventions to reduce state and trait anxiety in clinical settings should be tailored accordingly. Control-value theory of achievement emotions explains these patterns: emotions arise from students’ perceived control and task value. Moderate state anxiety can boost motivation and cognitive performance if control is sufficient, while excessive anxiety (state or trait) drains cognitive resources and impairs learning. Conversely, high satisfaction enhances positive learning appraisals, boosting motivation, engagement, and proactive refinement of technical skills. Nursing students who experience greater satisfaction are more likely to participate actively, seek constructive feedback, and build resilience and competence in clinical practice (52, 53).

### 14.2 Practical implications

This has implications for nursing practice and research. While active instructor engagement can help some students, educators should limit direct participation during examinations to create a psychologically supportive environment that facilitates authentic demonstration of knowledge and competencies, especially for re-demonstrations. Indirect observation, such as one-way mirrors, may reduce pressure and improve focus in clinical and simulation settings, but feasibility must be carefully weighed. Implementing one-way observation requires infrastructural investments (privacy-preserving monitoring) and comprehensive faculty training for timely, constructive feedback, as well as adjustments to resources and evaluation protocols. Pilot studies and systematic evaluations are needed to assess cost-effectiveness and impact on engagement, informing broader adoption across diverse nursing education contexts. Future research should also test replication in other health disciplines and consider a twofold simulation strategy. First, Adaptive simulation models tailor the experience to individual learners by dynamically adjusting challenge and pacing to match performance, reducing anxiety and improving learning satisfaction (54, 55). Second, Structured mentorship programs can reduce anxiety and boost performance among nursing students, providing personalized support and feedback that may address gender-based anxiety differences observed in related contexts (56, 57).

## 15 Recommendations for further research

Future studies could extend findings on indirect supervision in clinical simulation assessments by identifying specific traits and contexts where it benefits learning, such as individual personality, prior simulation experience, and task difficulty. Refining simulation exercises may enhance assessment and learning effectiveness. A qualitative study comparing in-the-room versus out-of-the-room supervision with a purposive sample could clarify impacts on anxiety, self-confidence, and satisfaction, addressing the limited evidence on faculty presence in control rooms. Future studies should account for students’ backgrounds and traits and consider larger, multi-site samples. Collaborative university studies could enable more complex designs (e.g., split-plot ANOVA) (58) to address within and between subject variability in repeated-measures or nested data contexts.

## 16 Conclusion

Our findings indicate that instructor presence during clinical simulations exerts nuanced effects on student anxiety, self-confidence, and satisfaction: while guidance can support learning, excessive visible presence may elevate state anxiety and hinder independent problem-solving, whereas a balanced reduction in direct presence appears to sustain engagement without compromising perceived supervision. Empirically grounded implications include adopting indirect observation methods, such as one-way mirrors, to reduce perceived intrusiveness and lower anxiety while preserving timely feedback and incorporating adjunct technological options video recordings and vetted virtual simulation platforms as complementary means to support skill rehearsal and reflection while mitigating stress when integrated with structured debriefing. Gender differences emerged in anxiety and satisfaction responses, suggesting that indirect observation and technology-enhanced simulations should be evaluated for differential effects across genders and diverse cohorts. Findings are context-specific, underscoring the need for replication across additional nursing programs to determine broader applicability; future work should explicitly test indirect observation across programs and genders, compare in-the-room versus control-room supervision, assess cost-effectiveness and feasibility, and examine cross-cultural validity to inform policy, curriculum design, and faculty development.

## Data Availability

The raw data supporting the conclusions of this article will be made available by the authors, without undue reservation.
